# Endoscopic and intrathecal management of intraventricular fungal infections: a case report of *Cladophialophora bantiana* and systematic review

**DOI:** 10.1186/s12879-026-12638-7

**Published:** 2026-01-23

**Authors:** Jan Oros, Radwan Takroni, Majid S. A. Aljoghaiman, Clifford Pierre, Daniel F. Brown, Marcus Czabanka, Stephen Monteith

**Affiliations:** 1 Swedish Neuroscience Institute, Department of Neurosurgery, Seattle, WA USA; 2 Department of Neurosurgery, Goethe University Frankfurt, University Hospital, Frankfurt am Main, HE Germany; 3 CellNetix Pathology and Laboratories, Seattle, WA USA; 4 King Faisal Medical City for Southern Regions, Abha, Saudi Arabia; 5 Surgery Department, College of Medicine, King Faisal University, Hofuf, Saudi Arabia

**Keywords:** *Cladophialophora bantiana*, Fungal ventriculitis, Neuroendoscopy, Central nervous system infection, Ommaya reservoir, Brain abscess

## Abstract

**Background:**

Intraventricular fungal infections are rare and associated with high morbidity and mortality. Diagnosis is often delayed due to nonspecific clinical and radiological findings, and treatment is complicated by limited cerebrospinal fluid (CSF) penetration of systemic antifungal agents. Neuroendoscopic visualization and intrathecal antifungal therapy have been reported only sporadically, and standardized management strategies are lacking.

**Case presentation:**

We report a 75-year-old male who developed a right temporal lobe abscess caused by *Cladophialophora bantiana*, confirmed by histopathology, culture, and molecular diagnostics. Despite prolonged systemic antifungal therapy, the patient demonstrated clinical and radiographic progression with intraventricular ventriculitis. Neuroendoscopy enabled direct visualization of pigmented intraventricular fungal lesions, targeted biopsy, ventricular lavage, and placement of an Ommaya reservoir, allowing initiation of intrathecal liposomal amphotericin B with serial CSF drug level monitoring and subsequent radiographic stabilization. To contextualize this case, we performed a systematic literature review of intraventricular fungal infections managed with endoscopic procedures and/or intrathecal or intraventricular antifungal therapy, identifying eleven cases involving *Aspergillus spp.*,* Cladophialophora bantiana*,* Mucorales*, and other dematiaceous fungi. Management strategies were highly individualized and typically combined surgical intervention, ventricular access devices, systemic antifungals, and local antifungal administration.

**Conclusion:**

This case report and systematic review highlight neuroendoscopy as a key diagnostic and therapeutic tool in intraventricular fungal infections and underscore the frequent need for intrathecal antifungal therapy when systemic treatment is insufficient. Management remains interdisciplinary and individualized, emphasizing the importance of detailed reporting to inform future treatment strategies.

**Clinical trial number:**

Not applicable.

**Supplementary Information:**

The online version contains supplementary material available at 10.1186/s12879-026-12638-7.

## Background

*Cladophialophora bantiana* is a rare, neurotropic, melanin-producing dematiaceous fungus that can lead to life-threatening brain abscesses and ventriculitis. While fungal CNS infections are generally associated with immunocompromised hosts, *C. bantiana* has the unique capacity to infect immunocompetent individuals, thereby complicating diagnosis and delaying treatment [1–3]. The fungus’s ability to survive in harsh microenvironments and evade immune defences contributes to its virulence. The mortality rate associated with central nervous system (CNS) phaeohyphomycosis, caused by *C. bantiana*, has been reported to exceed 65%, even in cases that have undergone aggressive antifungal therapy. The organism is typically acquired through inhalation, followed by hematogenous dissemination to the CNS. However, the initial pulmonary focus is often subclinical or undetectable [1].

Imaging modalities, such as magnetic resonance imaging (MRI) or computerised tomography (CT), may reveal abscess formations, however, a definitive diagnosis generally necessitates microbiological confirmation through biopsy or culture. Advancements have also been made in next-generation sequencing [2]. The implementation of endoscopic procedures for the visualisation of fungal infections within the CNS is exceedingly rare, with only handful of documented cases [3, 4].

In this particular instance, the case we present is notable for the use of neuroendoscopy to directly observe pigmented intraventricular lesions consistent with *C. bantiana*, providing both diagnostic material and a route for the administration of intrathecal therapy. Moreover, our patient was not in a chronically immunocompromised state and developed progressive ventriculitis following a COVID-related exacerbation of chronic pulmonary disease and corticosteroid exposure, which suggests a potential widening range of susceptibility in the context of emerging infections and global environmental shifts [1]. Due to the lack of guidelines and scarcity of recommendations for intraventricular and endoscopic management of CNS dematiaceous fungal infections, we conducted additional systematic review of literature:

## Methods of the systematic literature review

A systematic literature search was conducted in PubMed/MEDLINE, Embase, and Scopus for the period January 2000 to November 2025, restricted to full-text articles in English language. The search combined terms relating to fungal CNS infection, endoscopic or minimally invasive neurosurgical procedures, and ventricular involvement, using the following Boolean expression:

*(“Cladophialophora bantiana” OR phaeohyphomycosis OR “black fungi” OR “dematiaceous fungus” OR aspergillosis OR mucormycosis OR “fungal ventriculitis” OR “fungal brain abscess”) AND (endoscopic OR neuroendoscopic OR “ventricular lavage” OR “Ommaya reservoir” OR “intraventricular therapy” OR “minimally invasive” OR “stereotactic aspiration”) AND (“central nervous system” OR brain OR intracranial OR ventricle)*.

Reference lists of included papers were screened to identify additional eligible studies.

The review question was formulated using the PICO framework, defining the population as patients with intracranial fungal infections involving the ventricular system; the intervention as endoscopic procedures, stereotactic aspiration, or ventricular access devices used diagnostically or therapeutically. All identified records were screened for relevance, and full texts were assessed according to predefined eligibility criteria focusing on original patient data describing ventricular involvement and an endoscopic, stereotactic, or intrathecal/intraventricular treatment component. Studies without ventricular involvement, without fungal aetiology, or without an endoscopic or intrathecal component were excluded. In accordance with PRISMA 2020 principles, a structured selection process was followed.

## Case presentation

### Initial presentation and admission

**Fig. 1:**
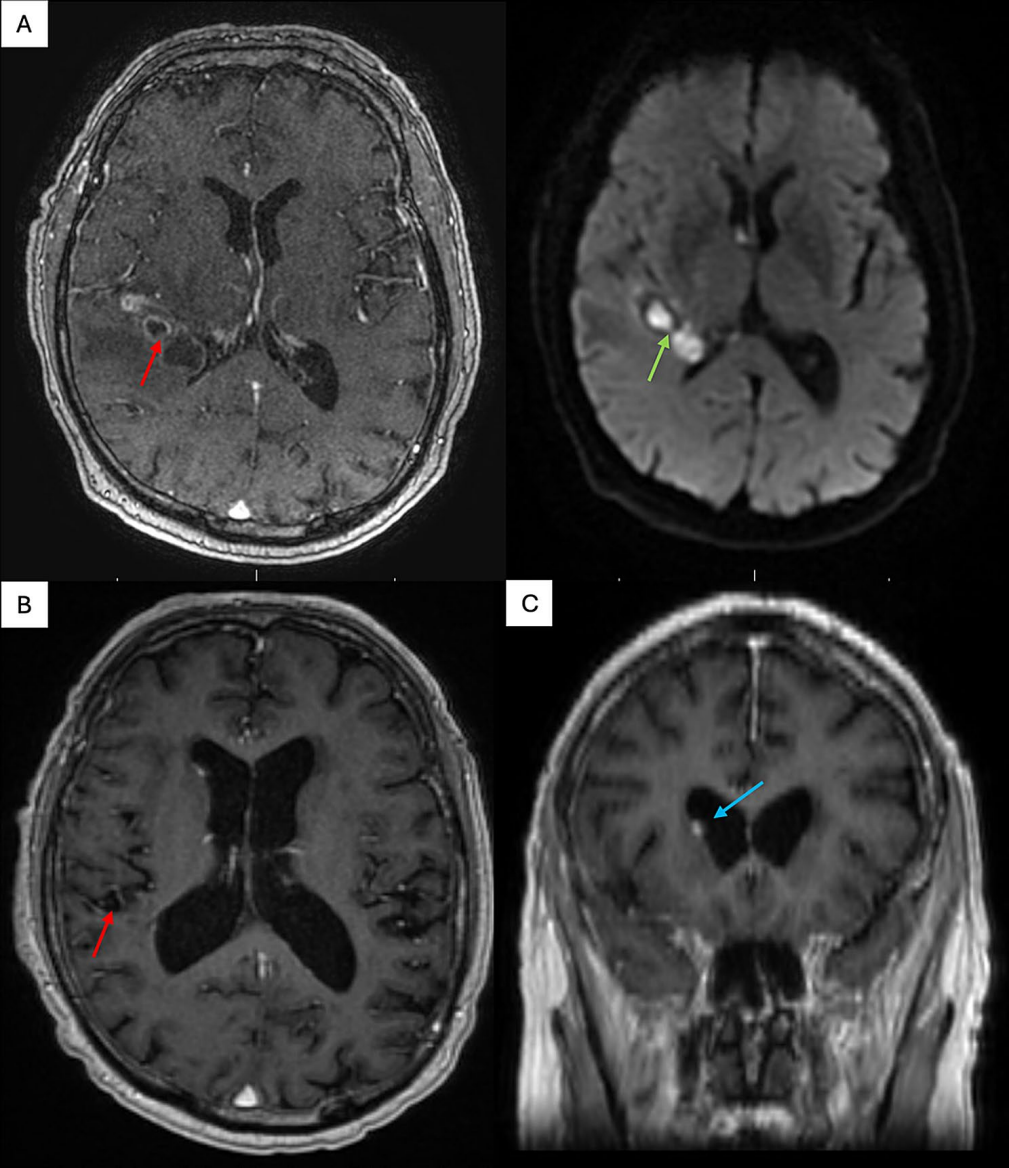
Preoperative MRI imaging. **A**: T1 weighed axial MRI sequence with contrast depicting multiple abscess-suspicious lesions adjacent to the posterior horn of the right lateral ventricle with marginal contrast enhancement (red arrow) and DWI axial sequence with characteristic diffusion restriction (green arrow). **B**: T1 weighed axial MRI sequence with contrast depicting significant reduction in abscess formation after surgical burr hole evacuation (red arrow). **C**: T1 weighed coronal MRI sequence with contrast depicting small ependymal lesion adjacent to the lateral wall of the right lateral ventricle (blue arrow). MRI = Magnetic Resonance Imaging; DWI = Diffusion Weight Imaging

A 75-year-old male patient was initially admitted with intermittent fevers and headaches. At the time of the initial presentation, various comorbidities were documented, including heart failure with reduced ejection fraction (HFrEF), arterial hypertension (aHTN), chronic obstructive pulmonary disease (COPD), atrial fibrillation on oral anticoagulation, chronic kidney disease (CKD) stage III, and a history of prostate cancer with status post radiation therapy. The patient lives north of Seattle, WA, USA (Pacific Northwest) and showed no occupational (retired) or travel hazards. The initial brain MRI scan revealed a peripherally enhancing lobulated lesion with decreased DWI signal within the right temporal lobe, suggesting the presence of a brain abscess (Fig. 1A).

### Initial management

The patient was initially treated with broad-spectrum intravenous antibiotics (Cefepime, Metronidazole & Vancomycin) due to suspected brain abscess of bacterial origin. This treatment was followed by a brain biopsy and burr hole aspiration of the lesion. A thorough pathological examination revealed features that were consistent with the presence of pigmented hyphae. Concurrently, both PCR and cultures yielded results that confirmed the presence of *C. bantiana*; leading to transition to the antifungal agents. Since no established breakpoints exist for *C. bantiana* in the antifungal susceptibility testing, the therapeutic approach with the highest CNS-permeability was selected. Initial course comprised of IV liposomal amphotericin B (L-AmB). Since this was purely tolerated due to patient’s CKD, L-AmB was discontinued after five days and replaced with PO voriconazole 400 mg BID until serum throughs reached therapeutic levels above 2 mg/L. This was reached on the fifth day of therapy, after which the maintenance dose of PO voriconazole of 200 mg BID was established and the patient was discharged to outpatient care.

### Outpatient follow-up and recurrence

In the six-month period following discharge, the adherence to the PO antifungal therapy was measured on multiple occasions, always showing therapeutic serum voriconazole concentrations (Fig. 5). A series of MRI follow-up scans were conducted, revealing no substantial reduction in the size of the lesions. However, there was a noticeable attenuation of perifocal oedema, with no concomitant increase in the size or number of lesions. Five months after the initiation of antimycotic therapy, the patient was admitted with a coinfection of the novel corona virus (SARS-CoV-2), which exacerbated his chronic obstructive pulmonary disease (COPD). This development necessitated the administration of a brief course of corticosteroid therapy, namely PO prednisolone 40 mg QD for 5 days. Three months after the COPD exacerbation and eight months after the diagnosis of the invasive CNS phaeohyphomycosis, the patient presented with increasing headaches, gait unsteadiness, and clumsiness. A cranial MRI scan was performed, revealing a stable parenchymal lesion (Fig. 1B). However, significant progression of ependymal lesions in the lateral ventricles was observed, indicating a deteriorating ventriculitis (Fig. 1C). Voriconazole through showed sufficient serum concentrations of 3.2 mg/L (Fig. 5). Due to the clinical and radiographic progression of the disease, the patient was indicated for an endoscopic ventricle lavage with biopsy of the suspected lesions, followed by the subsequent implantation of an Ommaya reservoir to facilitate the intrathecal administration of antimycotic agents.

### Intraoperative findings

**Fig. 2:**
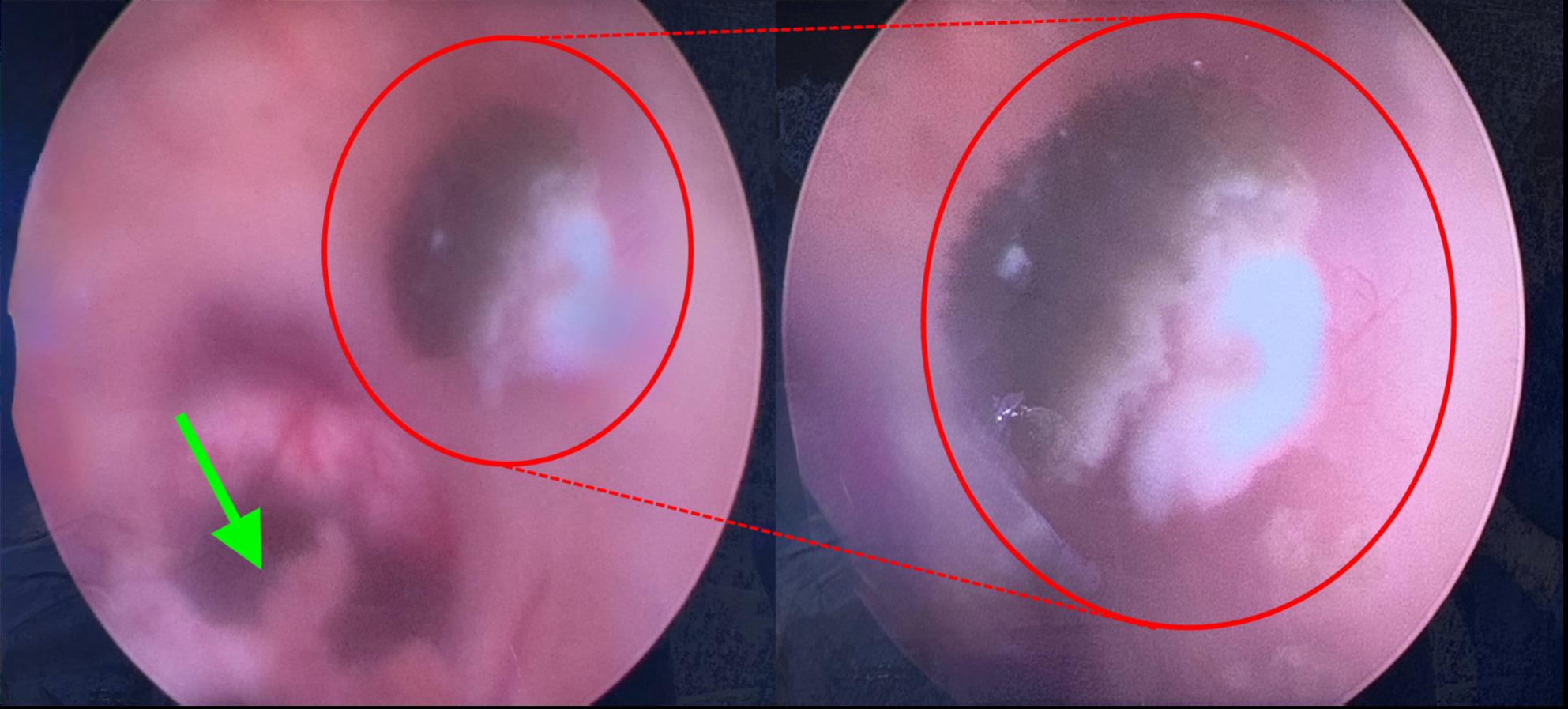
Endoscopic depiction of the intraventricular fungal filaments. Left: Endoscopic view of the lateral wall of the right lateral ventricle with choroid plexus (green arrow) near the foramen of Monro and darkly pigmented filaments of the *C. bantiana* firmly adhering to the wall of the lateral ventricle (smaller red circle). Right: Magnification on the darkly pigmented filaments of the *C. bantiana* (larger red circle)

**Fig. 3:**
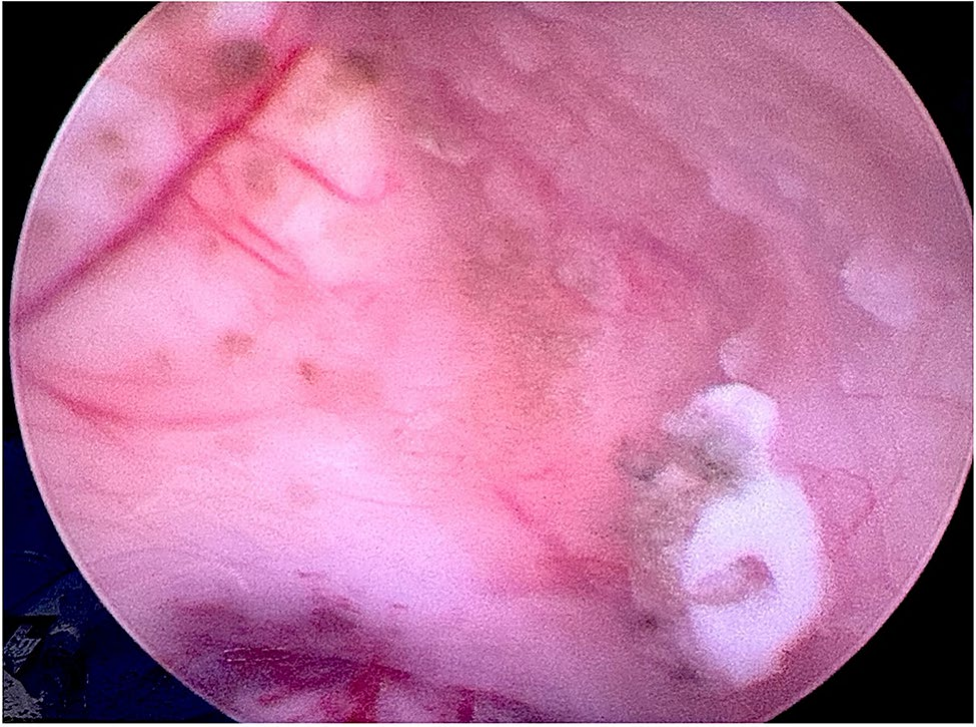
Endoscopic depiction of the lateral ventricle with the diffuse spread of *C. Bantiana.* Endoscopic view of the lateral wall of the right lateral ventricle with diffuse patches of pigmented filaments (red arrow) after the sampling of the most prominent lesion

After the navigated approach to the right lateral ventricle was established, an endoscope was inserted, accompanied by continuous lavage with Ringer solution. Matching the preoperative MRI findings (Fig. 1C), a fluffy, kidney shaped, darkly pigmented lesion adjacent just dorsolateral to the right foramen of Monro was identified (Fig. 2, Video 1). The endoscopic inspection of the right lateral ventricle also revealed the presence of additional diffuse darkly pigmented patches. For the subsequent histopathological and microbiological analysis, biopsy forceps were introduced intraventricularly, and the largest lesion was sampled (Video 2). Due to the diffuse spread of fungal foci (Fig. 3) in the lateral ventricle, the complete removal of the fungal foci was not feasible. After lavaging the ventricle, the endoscope was removed, followed by Ommaya reservoir implantation.

### Postoperative work up and outpatient follow-up

**Fig. 4:**
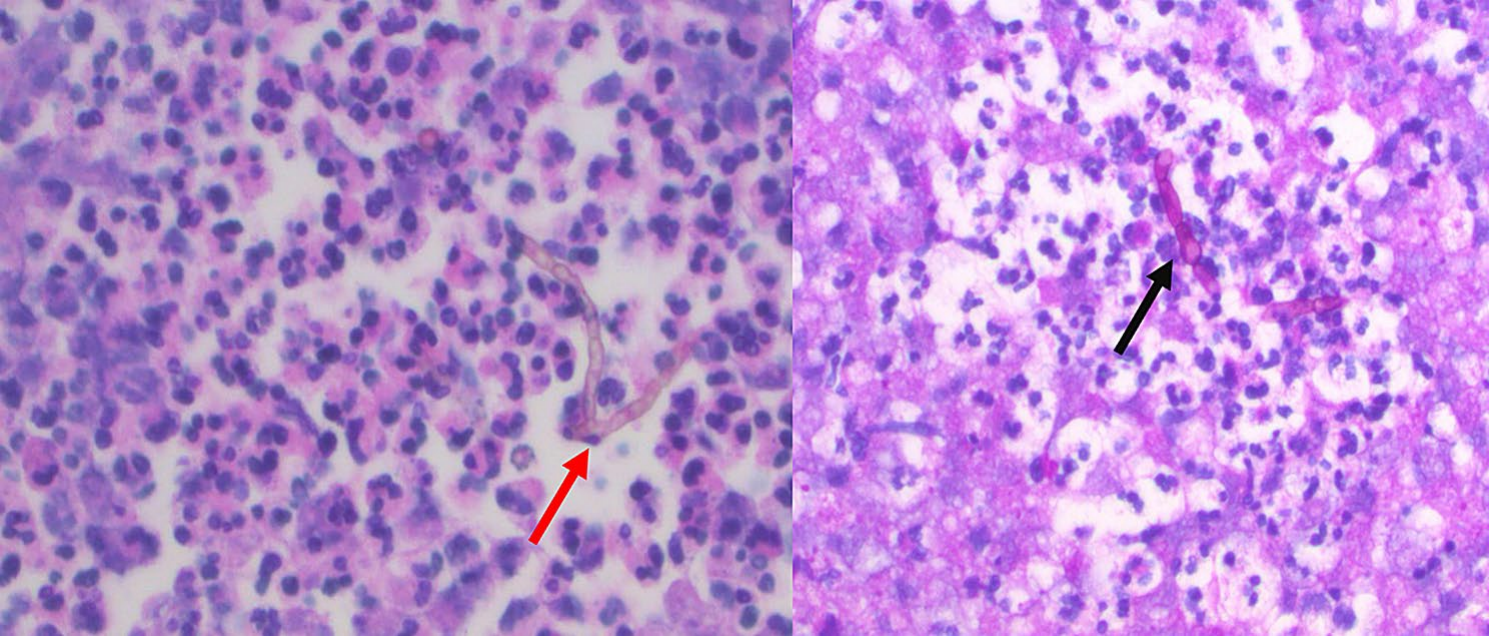
Microscopic depiction of the septate hyphae of *C. Bantiana.* Left: H&E-stained section showing the characteristic pigmented unbranched septate hyphae (red arrow) in a wavy chain amongst a background of neutrophils at 400x magnification. Right: PAS stain highlighting the hyphae in dark red (black arrow) at 400x magnification. H&E = Hematoxylin & Eosin, PAS = Periodic Acid-Schiff

**Fig. 5:**
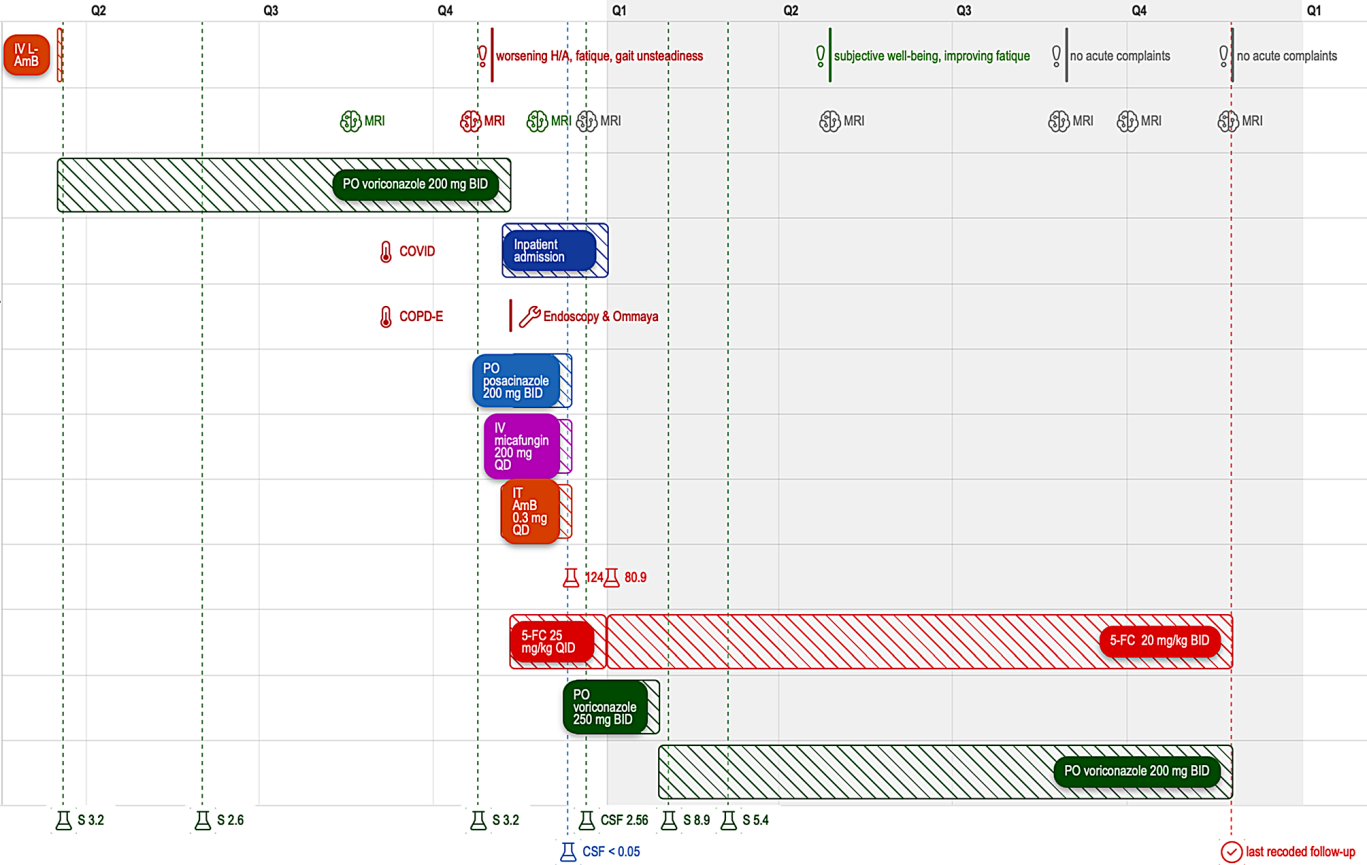
Graphic timeline^*^ of the disease course. Timeline of clinical course, imaging (*brain icon; green = improvement; red = progressive disease; grey = stable disease*), antifungal therapy, S- and CSF-monitoring in mg/L (*lab vial icon; red = 5-FC; green = voriconazole*,*; blue = posaconazole*); and clinical follow-ups (*exclamation sign; green = improvement; red = new symptoms; grey = stable disease*). Shown are periods of IV and oral antifungal treatments, intrathecal therapy, clinical events (e.g., headache worsening, gait unsteadiness, COVID, COPD exacerbation), hospital admission with Ommaya placement, and serial MRI/CSF assessments. Serum and CSF antifungal levels are indicated where available. The dashed red line marks the last recorded follow-up (*check sign*). IV = intravenous; L-AmB = liposomal amphotericin B; PO = per oral; BID = twice daily; QD = once daily; QID = four times daily; IT = intrathecal; 5-FC = flucytosine; COPD-E = COPD exacerbation; MRI = magnetic resonance imaging; CSF = cerebrospinal fluid; S = serum level; H/A = headache. *The timeline was created using the Superjane app (https://www.superjane.app)

The intraoperatively collected tissue was cultured on Sabouraud dextrose agar (with and without antibiotics) and incubated at 30 °C and 37 °C. Growth was slow, with colonies appearing after several days. The mold demonstrated thermotolerance with growth at 37 °C, a key feature for neurotropic dematiaceous fungi. Panfungal PCR targeting the internal transcribed spacer (ITS) region (ITS1–ITS2) of ribosomal DNA, confirming the presence of *C. bantiana*. H&E staining showed septate hyphae with brown pigmented cell walls, consistent with a dematiaceous fungus, whereas PAS staining highlighted the fungal cell walls, confirming the presence of septate hyphae consistent with *C. bantiana*. (Fig. 4, left and right). Based on results of antifungal susceptibility testing, the patient was treated with quadruple antifungal therapy (5-FC at an initial dose of 25 mg/kg QID, IV micafungin 200 mg QD, PO posaconazole 200 mg BID and intrathecal amphotericin B). Due to high concentrations of serum 5-FC (124 mg/L), the dose was adjusted to 20 mg/kg BID prior to discharge, after which the concentrations returned to therapeutic levels (80.9 mg/L). Repeat brain MRIs noted findings consistent with improving ventriculitis and right temporal lobe abscess with stable changes of ventriculitis. After the patient had completed one month of quadruple antifungal therapy, he was transitioned to dual therapy with 5-FC at 20 mg/kg BID and posaconazole at 200 mg BID. Due to undetectable posaconazole levels in the CSF on follow-up (< 0.05 mg/L); measured using modified liquid chromatography-tandem mass spectrometry (LC-MS/MS assay) [5, 6], posaconazole was later transitioned back to voriconazole at 250 mg BID and later reduced to 200 mg BID due to high serum concentrations (8.9 mg/L). Relevant check points in the course of the disease are depicted in a graphic timeline (Fig. 5).

**Fig. 6:**
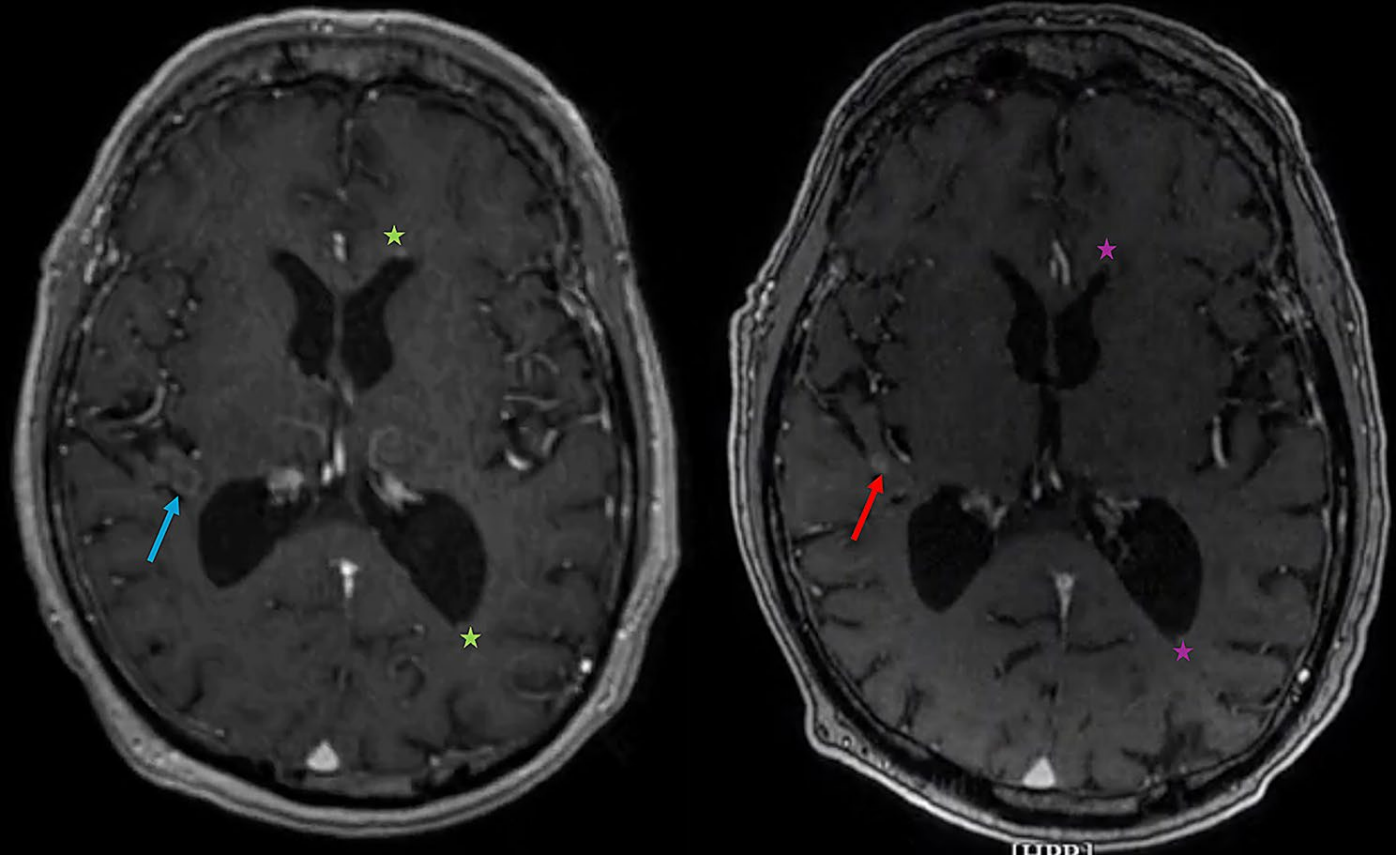
Postoperative and follow-up MRI imaging. Comparison of the postoperative T1-weighed post-contrast axial MRI sequence prior to discharge of the patient (left) with the one-year-follow-up T1-weighed post-contrast axial MRI sequence. The residual perilesional contrast enhancement on the postoperative imaging (blue arrow) shows signs of regression and consolidation on one-year-follow-up (red arrow). Patches of contrast enhancement in the anterior and posterior horns of lateral ventricles that were seen on the postoperative imaging (green asterix) are also consistent with regressing ventriculitis on the one-year-follow-up MRI (violet asterix). MRI = Magnetic Resonance Imaging

Multiple outpatient visits followed including serial repeat MRI follow-ups. Except of intermittent headaches, the patients complained of no further symptoms during this period. The serial MRI follow-ups showed stable scattered small periventricular enhancing nodules consistent with stable ventriculitis with no new abnormalities (Fig. 6).

## Results of the systematic literature review

The systematic search yielded 124 records, of which 31 full-text articles were reviewed in detail. Twenty-three were excluded for not meeting eligibility criteria, leaving eight studies, one of which contained three cases. Including our own case, the final dataset comprised 11 patients. Because all included studies were case reports, quantitative synthesis and risk-of-bias assessment were not applicable. Data on demographics, fungal species, surgical management, antifungal regimens (intravenous and intrathecal), treatment duration, timing, and outcomes were extracted manually and synthesized descriptively (Tables 1 and 2).

**Table 1:** Basic characteristics of the studies included in the systematic literature review

Publication year	Study	Demographics, comorbidities & hazards	Isolated fungus	Surgical approach	Outcome
2003	Raut et al.	26 yo female immunosuppression SLE on long-term steroid use	C. bantiana	Burr hole aspiration → craniotomy	Radiographic progress after 2 weeks, clinical recovery with neurological sequelae (right hemiparesis) at 24 months
2008	Metellus at al.	42 yo female immunocompetent IV drug abuse	Mucorales spp.	Endoscopic debridement & Ommaya	Complete radiographic remission, partial clinical recovery (left arm paresis) at 3 years
2010	Wang et al.	39 yo male immunocompetent schizophrenia	Aspergillus spp.	Endoscopic debridement & EVD → temporary VPS	Complete radiographic & clinical recovery w/o sequelae
2016	Kantarcioglu et al.	28 yo male immunocompetent coal mine worker	C. bantiana	2x burr hole aspiration → VPS → Ommaya	Death at 2 years
2020	Schauwvlieghe et al. Case 1	18 yo female immunosuppression ALL	A. fumigatus	Ommaya	Partial radiographic remission, partial clinical recovery (mild arm paresis) at 4 months
	Case 2	16 yo female immunosuppression β-thalassemia after allo-SCT	A. fumigatus	Ommaya	Partial radiographic remission, severe disability at 9 years
	Case 3	15 yo female immunosuppression ALL	A. fumigatus	Ommaya	Complete radiographic remission, clinical recovery w/o sequelae at 8 months
2023	Sahoo et al.	29 yo male immunocompetent IV drug abuse	N/R	Endoscopic excision & septostomy	Death at 5 months
2024	Nguyen et al.	23 yo male immunocompetent IV drug abuse	Rhizopus spp.	EVD → ETV & endoscopic biopsy	Complete radiographic remission, clinical recovery w/o sequelae
2024	Çaglar Kizil et al.	12 yo male immunosuppression renal transplant	A. fumigatus	2x Burr-hole drainage → Ommaya	Minimal radiographic remission at 3 months, clinical recovery w/o sequelae at 8 months
2025	Oros et al.	75 yo male immunocompetent short-term corticoid use	C. bantiana	Endoscopic biopsy, lavage & Ommaya	Radiologic & clinical improvement

yo = years old, IV = intravenous; EVD = external ventricle drain; VPS = ventriculo-peritoneal shunt; w/o = without; allo-SCT = allogenic stem cell transplantation; ALL = acute lymphoblastic leukemia; N/R = not reported; ETV = endoscopic third ventriculostomy

**Table 2:** Therapeutic regimens accross the studies included in the systematic literature review

Year	Authors	Systemic regimens (doses)	Duration [weeks]	IT/IL regimens (doses)	Duration [weeks]	Time to initiation of IT/IL regimen [week]
2003	Raut et al.	L-AmB (40 mg cumulative)	N/R	L-AmB (0.5 mg/week)	2	1
2008	Metellus et al.	L-AmB (5 mg/kg QD)	26	L-AmB (0.5 mg QID)	12	1
2010	Chen et al.	L-AmB (N/R) IV voriconazole (200 mg BID)	2 N/A	None	N/A	N/A
2016	Kantarcioglu et al.	L-AmB (5 mg/kg QD) IV voriconazole (6 → 4 mg/kg/day)	26 25	L-AmB (1 mg QD)	19	21
2020	Schauwvlieghe et al. Case 1	L-AmB (3–10 mg/kg QD); IV voriconazole (8 mg/kg BID); PO posaconazole (300 mg BID)	45 1 27	L-AmB (1 mg/week)	16	51
	Case 2	L-AmB (6 mg/kg QD); IV voriconazole (8 mg/kg BID); IV caspofungin (70 mg QD); PO 5-FC (25 mg/kg QID)	29 1 27 15	L-AmB (1 mg/week); caspofungin (1 mg QD; → 1 mg 3x/week; → 0.5 mg 3x/week)	10 2 4 21	4 2
	Case 3	L-AmB (5 mg/kg/QD); IV voriconazole (4 mg/kg BID); isavuconazole (N/R)	32 25 N/R	L-AmB (1 mg/week)	32	3
2023	Sahoo et al.	L-AmB (N/R)	N/R	None	N/A	N/A
2024	Nguyen et al.	L-AmB (N/R) isavuconazole (N/R)	1 3	L- AmB (N/S)	3	1
2024	Franzese et al.	L-AmB (N/R); IV voriconazole (N/R)	40 N/R	L-AmB 1 mg/week → 3x/week	20 12	N/R
2025	Oros et al.	L-AmB 5 mg/kg QD; PO voriconazole (200 mg BID; → 250 mg BID; → 200 mg BID) micafungin 200 mg QD PO 5-FC (25 mg/kg QID; → 20 mg/kg QID) PO posaconazole 200 mg BID	1 34 7 43 4 4 50 4	L-AmB (0.3 mg QD)	4	32

IV = intravenous; IT/IL = intrathecal/intralesional; L-AmB = liposomal amphotericin B; N/R = not reported; N/A = not applicable

### Basic characteristics

Most patients were adults (8/11; 72.7%) [7–13], whereas paediatric cases were represented by three patients. Six of 11 patients (54.5%) were classified as immunocompetent at presentation [8–10, 12, 13], although four of these had clear predisposing factors for opportunistic fungal infection: intravenous drug abuse in three cases [8, 12, 13], and occupational coal dust exposure in one case [10]. Short-term corticosteroid use in our patient represented an additional transient risk factor. The remaining five patients (45.5%) were immunosuppressed acute lymphoblastic leukaemia (two patients), ß-thalassemia after allogeneic stem-cell transplantation [11], systemic lupus erythematosus [7] and renal transplantation [14].

The most frequently isolated organism was *Aspergillus fumigatus/Aspergillus spp*., identified in 5/11 cases (45.5%) [9, 11, 14]. *C. bantiana* was documented in three cases (27.3%) [7, 15]. Following fungal species were only isolated in single cases: *Mucorales* [8] and *Rhizopus spp.* [13]. In the case reported by Sahoo et al., the diagnosis was phaeohyphomycosis but the exact species was not specified [12]. All basic characteristics are summarised in Table 1.

### Surgical management

No uniform surgical concept emerged across the 11 cases. As illustrated in Table 1, neuroendoscopic techniques played a central role in several reports, including our own [8, 9, 12, 13]. Only in two case reports, including ours, this was combined with the implantation of an Ommaya reservoir for intrathecal antifungal application Wang et al. [8] placed an external ventricle drain (EVD) after endoscopic debridement, which was followed by the implantation of a temporary ventriculo-peritoneal shunt (VPS) to address the temporary CSF flow disturbance, without intrathecal antifungal therapy [9]. Sahoo et al. performed endoscopic excision and septostomy for hydrocephalus, but did not use intrathecal antifungal agents [12] In contrast, Nguyen et al. combined endoscopic third ventriculostomy and biopsy with EVD placement, which was then used for short-term intrathecal therapy [13]. The placement of Ommaya reservoir was used in 7 of 11 patients (63.6%) either as the primary surgical approach [11], or as an adjunct to endoscopic debridement [8, 13] as in our case, or to burr hole drainage [10, 14]. Despite heterogeneity, a clear pattern emerges: most authors favoured establishing durable ventricular access (Ommaya, EVD or VPS) both for CSF management and for potential intrathecal (intraventricular) drug delivery. Further details of surgical therapy are depicted in Table 1.

### Management of antifungal treatment

In the vast majority of the patients (9/11; 81.1%) received intrathecal or intraventricular (IT) antifungal therapy, administered via Ommaya reservoir, EVD, VPS, or directly into the lesion cavity (intralesional therapy) [8, 11, 13–15]. L-AmB was the agent of choice for IT/IL administration in all but one patient, in whom L-AmB was combined with intrathecal caspofungin initially at 1 mg/day with reduction to 1 mg 3x/weeks and 0.5 mg 3x/week after 2 and 4 weeks respectively [11]. There was a significant spread between administered dose of the intrathecal therapy. From doses of IT L-AmB as low as 0.5 mg/week [7] through 1 mg/day [11] to as high as 1 mg/day [10] were reported.

The timing and duration of IT/IL therapy varied markedly. Only in three cases, the intrathecal therapy was initiated within the first week after diagnosis [7, 8, 13]. At the opposite extreme, intraventricular therapy in the β-thalassemia patient reported by Schauwvlieghe et al. was initiated 51 weeks after diagnosis of intracranial fungal infection, following failure of systemic therapy [11]. Across all cases with available data, the duration of IT/IL therapy ranged from 2 to 32 weeks with a median of 16 weeks. In most reports, local therapy was well tolerated; only one patient required discontinuation after three weeks due to severe headache [13].

Systemic antifungal regimens are summarised in Table 2. L-AmB at doses between 3 and 10 mg/kg/day was the backbone of intravenous therapy in nearly all cases, frequently in combination with triazoles (voriconazole 4–8 mg/kg twice daily or posaconazole 300 mg twice daily) and, in selected patients, flucytosine and caspofungin [11]. Voriconazole was often discontinued or escalated due to insufficient clinical response despite therapeutic serum levels, including in our patient [11, 14, 15]. Isavuconazole was used as a later-line systemic agent in some cases [11, 13]. Nephrotoxicity emerged as the most common reason for stopping systemic L-AmB, which was also the case in our patient [13].

### Outcomes

Across the 11 included patients, 9 (81.8%) survived their initial episode of intracranial fungal infection, although the degree of neurological recovery varied. Two patients (18.2%) died, one with invasive *C. bantiana* infection [10] and one with intraventricular phaeohyphomycosis [12]. Among survivors, outcomes ranged from complete or near-complete recovery to persistent neurological deficits. The dataset shows considerable heterogeneity, and because the reports differ substantially in fungal species, immune status, extent and localization of disease, and treatment strategies, no clear association can be established between survival and any individual factor such as timing of intrathecal therapy, choice of antifungal agent, or surgical approach. Localization (e.g., diffuse ventricular vs. focal parenchymal involvement) appears to vary widely among cases but cannot be quantitatively linked to prognosis based on available evidence. A case-by-case summary of outcomes is provided in Table 1.

## Discussion and conclusions

Intracranial fungal infections involving the ventricular system treated with neuroendoscopy and/or intrathecal antifungal therapy are exceptionally rare, and consequently, the available evidence is limited to isolated case reports. The eleven identified cases including ours represent a heterogeneous group with respect to patient demographics, underlying immune status, fungal species, anatomical patterns of disease, and therapeutic strategies. This heterogeneity precludes the development of generalized treatment algorithms but allows several descriptive observations.

We described a rare recurrent intracerebral and intraventricular infection caused by *C. bantiana* in a formally immunocompetent patient, including direct endoscopic visualization of intraventricular fungal lesions. In the following review, ventricular involvement occurred across a broad immunological spectrum. Although nearly half of the patients were immunosuppressed due to hematologic disease, systemic therapy, or transplantation, a substantial proportion were formally immunocompetent, often with only transient or indirect risk factors such as intravenous drug use, short corticosteroid exposure, or occupational environmental contact. This supports the notion that ventricular fungal disease is not confined to severely immunocompromised populations [15]. Whether transient immunomodulation due to COVID-19 infection and corticosteroid use due to COPD-exacerbation in our patient contributed to disease progression remains uncertain, although similar associations have been reported in other cases of *C. bantiana* infections [16, 17]. No pulmonary fungal focus was identified in our patient.

The fungal species varied widely, spanning *Aspergillus spp.*, *C. bantiana*, *Mucorales spp.* and *Rhizopus spp*. Despite these microbiological differences, the clinical presentations and disease courses showed considerable overlap, with headache, focal deficits, and radiological evidence of obstructive or ependymal disease being common features. The diversity of pathogens further underscores the need for broad diagnostic consideration in atypical intraventricular or multiloculated lesions.

Following initial surgical excision, our patient received systemic voriconazole and L-AmB. Despite evidence supporting CSF penetration of voriconazole, radiographic progression occurred eight months later. Endoscopic lavage and Ommaya placement allowed initiation of intrathecal L-AmB and facilitated combination therapy with posaconazole, micafungin, and flucytosine. Similar multimodal approaches were variably reported in the cases included in the review, although detailed endoscopic descriptions and pharmacokinetic monitoring were rarely provided.

Surgical management strategies also differed considerably. Approximately half of all cases employed neuroendoscopic procedures for diagnosis or partial debridement, and in many instances, ventricular access devices such as Ommaya reservoirs or external ventricular drains were placed to enable CSF diversion, repeated sampling, or local antifungal administration. These approaches were used not according to fixed criteria but rather as pragmatic responses to ventricular obstruction, diagnostic uncertainty, or the need for intrathecal therapy. No single surgical strategy emerged as dominant or consistently associated with superior outcomes, reflecting individualized decision-making driven by lesion location, disease extent, and comorbidity profile.

Antifungal therapy was similarly heterogeneous. L-AmB served as the cornerstone of both intravenous and intrathecal regimens, often in combination with triazoles, echinocandins, or flucytosine. However, dosing schedules, duration, and timing of intrathecal administration varied widely, and reporting was inconsistent across cases. Importantly, the review does not allow any inference regarding the relative efficacy of different antifungal combinations or routes of delivery. Intrathecal therapy was used in most cases, but its timing ranged from early initiation to late introduction after systemic treatment failure, with no discernible relationship to outcome. Current recommendations, largely based on small series, favour maximal safe resection when feasible and systemic antifungals with the best achievable CNS penetration. Nevertheless, the available data on CSF penetration of posaconazole vary largely [18, 19], mirrored by undetectable CSF concentrations in our patient and necessitating treatment modification. Due to inconsistent distribution in CSF, these agents are often administered in combination with or subsequent to L-AmB [20–22]. There are no specific guidelines in place for endoscopic procedures or intrathecal administration of antimycotic agents.

Geographical patterns of *C. bantiana* infection historically suggest a predominance in tropical and subtropical regions [15]; however, recent reports, including our case from the Pacific Northwest, indicate that cases increasingly occur in temperate climates without travel history. Environmental and climatic changes may contribute to this shifting epidemiology.

Outcomes varied from complete recovery to persistent neurological deficits or death. Two patients died, underscoring the severity of ventricular fungal infections, yet the small sample size and inconsistent follow-up limit prognostic interpretation. Based on the available data, no consistent association can be drawn between fungal species, immune status, surgical strategy, or timing of intrathecal therapy and clinical outcome.

The cases analysed in this review highlight several consistent themes rather than definitive clinical guidance. Ventricular fungal infections are rare, diagnostically challenging, and managed through individualized combinations of surgical and antifungal strategies. Neuroendoscopy and ventricular access devices may aid in diagnosis and allow targeted therapy, but their use is guided by case-specific anatomical and clinical considerations rather than evidence-based criteria. Intrathecal or intraventricular L-AmB is frequently employed, yet optimal dosing and duration remain undefined. These findings underscore the need for more systematic reporting of clinical details, pharmacokinetic data, and long-term outcomes to inform future management recommendations.

## Supplementary Information

Below is the link to the electronic supplementary material.

Supplementary Material 1: Video 1. Intraoperative neuroendoscopic view: Intraoperative neuroendoscopic view showing pigmented fungal lesions within the right lateral ventricle adjacent to the foramen of Monro

Supplementary Material 2: Video 2. Endoscopic biopsy: Endoscopic biopsy of intraventricular lesion and lavage with Ringer’s solution prior to Ommaya reservoir placement

## Data Availability

The data supporting our findings are available, but restrictions apply to the availability of these data, which were used under license for the current study, and so are not publicly available. Data are however available from the authors upon reasonable request and with permission of the above-mentioned patient.

## Declarations

### Implementation of LLM declaration

DeepL (LLM) was used to improve general readability and stylistics of the manuscript. No original text was generated by the AI.

## Abbreviations

aHTN: Arterial hypertension
ALL: Acute lymphoblastic leukaemia
AmB: Amphotericin B
BID: Twice daily
CKD: Chronic kidney disease
CNS: Central nervous system
COPD: Chronic obstructive pulmonary disease
COPD-E: Chronic obstructive pulmonary disease exacerbation
CSF: Cerebrospinal fluid
CT: Computed tomography
DWI: Diffusion-weighted imaging
EVD: External ventricular drain
H&E: Hematoxylin and eosin
HFrEF: Heart failure with reduced ejection fraction
IL: Intralesional
IT: Intrathecal
ITS: Internal transcribed spacer
IV: Intravenous
LC-MS/MS: Liquid chromatography–tandem mass spectrometry
L-AmB: Liposomal amphotericin B
LSU: Large subunit ribosomal RNA
MRI: Magnetic resonance imaging
PAS: Periodic acid–Schiff
PCR: Polymerase chain reaction
PO: Per os (oral)
PRISMA: Preferred Reporting Items for Systematic Reviews and Meta-Analyses
QD: Once daily
QID: Four times daily
S: Serum
SLR: Systematic literature review
VPS: Ventriculoperitoneal shunt
5-FC: Flucytosine

## References

[1] Lortholary O, Garcia-Hermoso D, Sturny-Leclère A, Sitbon K, Nourrisson C, Letscher-Bru V, et al. Reappraising cladophialophora Bantiana phaeohyphomycosis in france: retrospective nation-based study. Lancet Microbe. 2024;5(11):100907. 39395429 10.1016/S2666-5247(24)00139-3

[2] Whitman M, Vissichelli N. A rare case of *Cladophialophora bantiana* intracranial infection: highlighting the utility of next-generation sequencing in diagnosis. Case Rep Transplant. 2024;(1):8892177. 10.1155/2024/8892177 PMC11455592 39372424

[3] Hagel S, Ewald C, Doenst T, Sachse S, Roedel J, Pletz MW. Ventriculitis due to infection with rhizopus arrhizus. Med Mycol Case Rep. 2015;10:18–20. 26862476 10.1016/j.mmcr.2015.12.004 PMC4706622

[4] Kankam SB, Saffar H, Shafizadeh M, Afhami S, Khoshnevisan A. Intraventricular CNS aspergillosis in a patient with prior history of COVID-19: Case report and review of literature. Ann Med Surg [Internet]. 2022 Aug [cited 2025 Apr 25];80. Available from: https://journals.lww.com/10.1016/j.amsu.2022.104122. 10.1016/j.amsu.2022.104122 PMC9259190 35821741

[5] Müller C, Gehlen D, Blaich C, Prozeller D, Liss B, Streichert T, et al. Reliable and Easy-To-Use liquid Chromatography–Tandem mass spectrometry method for simultaneous analysis of Fluconazole, Isavuconazole, Itraconazole, Hydroxy-Itraconazole, Posaconazole, and voriconazole in human plasma and serum. Ther Drug Monit. 2017;39(5):505–13. 28742650 10.1097/FTD.0000000000000438

[6] Körholz K, Holterhus M, Gordon K, Müller-Ohrem C, Müller C, Groll AH. Cerebrospinal fluid concentrations of posaconazole in paediatric leukaemia patients. J Antimicrob Chemother. 2024;79(3):564–6. 38198576 10.1093/jac/dkae005

[7] Raut A, Muzumdar D, Narlawar R, Nagar A, Ahmed N, Hira P. Cerebral abscess caused by cladosporium bantianum Infection-Case Report-: —Case Report—. Neurol Med Chir (Tokyo). 2003;43(8):413–5. 12968811 10.2176/nmc.43.413

[8] Metellus P, Laghmari M, Fuentes S, Eusebio A, Adetchessi T, Ranque S, et al. Successful treatment of a giant isolated cerebral mucormycotic (zygomycotic) abscess using endoscopic debridement: case report and therapeutic considerations. Surg Neurol. 2008;69(5):510–5. 17707491 10.1016/j.surneu.2007.02.035

[9] Wang YC, Wang SW, Cia CT, Chen PL, Shih HI, Choi PC, et al. Pneumonia and brain abscess likely due to cladophialophora Bantiana in a patient with systemic lupus erythematosus in Taiwan. J Microbiol Immunol Infect. 2024;57(1):204–6. 38171981 10.1016/j.jmii.2023.12.008

[10] Kantarcioglu AS, Guarro J, De Hoog GS, Apaydin H, Kiraz N, Balkan II, et al. A case of central nervous system infection due to cladophialophora Bantiana. Rev Iberoam Micol. 2016;33(4):237–41. 27453395 10.1016/j.riam.2016.01.004

[11] Schauwvlieghe AFAD, Bredius RGM, Verdijk RM, Smiers FJW, Van Der Beek MT, Goemans BF, et al. Management of cerebral azole-resistant *Aspergillus fumigatus* infection: a role for intraventricular liposomal-amphotericin B. J Glob Antimicrob Resist. 2020 Sep;22:354–7. 10.1016/j.jgar.2020.03.016 32251868

[12] Sahoo SK, Kaur H, Singh K. Primary intraventricular phaeohyphomycosis: an atypical presentation of neurotropic fungus. World Neurosurg. 2023;171:104–7. 36584890 10.1016/j.wneu.2022.12.099

[13] Nguyen KN, Freeman LM, Ung TH, Ojemann S, Grassia F. Immunocompetent isolated cerebral mucormycosis presenting with obstructive hydrocephalus: illustrative case. J Neurosurg Case Lessons. 2024;7(13):CASE23672. 38531080 10.3171/CASE23672 PMC10971069

[14] Çağlar Kizil HB, Tural Kara T, Çetin HS, Tekeli O, Açik AK, Kaya Aksoy G, et al. Central nervous system aspergillosis in a child after renal transplantation successfully treated with intraventricular therapy. Pediatr Infect Dis J. 2024;43(12):e472–3. 39163357 10.1097/INF.0000000000004513

[15] Kantarcioglu AS, Guarro J, De Hoog S, Apaydin H, Kiraz N. An updated comprehensive systematic review of *Cladophialophora bantiana* and analysis of epidemiology, clinical characteristics, and outcome of cerebral cases. Med Mycol. 2016:myw124. 10.1093/mmy/myw124 28007938

[16] Martínez-Lamas L, Álvarez M, Llovo J, Gené J, Cano J. Phaeohyphomycosis caused by *Cladophialophora bantiana*. Rev Iberoam Micol. 2014 July;31(3):203–6. 10.1016/j.riam.2013.05.004 23727472

[17] Revankar SG, Sutton DA, Rinaldi MG. Primary central nervous system phaeohyphomycosis: A review of 101 cases. Clin Infect Dis. 2004;38(2):206–16. 14699452 10.1086/380635

[18] Ruping MJGT, Albermann N, Ebinger F, Burckhardt I, Beisel C, Muller C et al. Posaconazole concentrations in the central nervous system. J Antimicrob Chemother. 2008 Sep 10;62(6):1468–70. 10.1093/jac/dkn409 18824458

[19] Reinwald M, Uharek L, Lampe D, Grobosch T, Thiel E, Schwartz S. Limited penetration of posaconazole into cerebrospinal fluid in an allogeneic stem cell recipient with invasive pulmonary aspergillosis. Bone Marrow Transpl. 2009;44(4):269–70. 10.1038/bmt.2009.17 19219077

[20] Kilbourn KJ, Green J, Zacharewski N, Aferzon J, Lawlor M, Jaffa M. Intracranial fungal *Cladophialophora bantiana* infection in a nonimmunocompromised patient: A case report and review of the literature. Surg Neurol Int. 2022;13:165. 35509580 10.25259/SNI_116_2022 PMC9062923

[21] Suri P. Cerebral phaeohyphomycosis due to *Cladophialophora bantiana* – a case report and review of literature from India. J Clin Diagn Res [Internet]. 2014 [cited 2025 Apr 25]; Available from: http://www.jcdr.net/article_fulltext.asp?issn=0973-709x%26year=2014%26volume=8%26issue=4%26page=DD01%26issn=0973-709x%26id=4216. 10.7860/JCDR/2014/7444.4216 PMC4064842 24959445

[22] Chowdhary A, Meis JF, Guarro J, De Hoog GS, Kathuria S, Arendrup MC, et al. ESCMID and ECMM joint clinical guidelines for the diagnosis and management of systemic phaeohyphomycosis: diseases caused by black fungi. Clin Microbiol Infect. 2014;20:47–75. 24483780 10.1111/1469-0691.12515

